# Effect of timing of surgery on postoperative complications and prognosis in elderly patients with hip fractures

**DOI:** 10.3389/fmed.2025.1646938

**Published:** 2025-10-01

**Authors:** Zuobin Zhuo, Weijun Hong, Guangxi Ma

**Affiliations:** ^1^Department of Orthopedic, Pingyang Hospital of Wenzhou Medical University, Wenzhou, China; ^2^Department of Orthopaedics, Wenzhou Hospital of Integrated Traditional Chinese and Western Medicine, Wenzhou, China

**Keywords:** timing of surgery, hip fracture, postoperative complications, Harris scores, prognosis

## Abstract

**Background:**

In clinical practice, there is no standardized criteria for the optimal timing of hip fracture surgery in the elderly, and there is much controversy.

**Objectives:**

To investigate the effect of timing of surgery on postoperative complications and prognosis in elderly patients with hip fractures.

**Methods:**

Retrospectively analyzed 636 elderly hip fracture patients over 65 years old. The patients were divided into early group (< 3 days), intermediate group (3–7 days) and late group (> 7 days) according to the time from fracture to surgery, and the three groups were compared with the postoperative in-hospital general conditions, the occurrence of complications, the efficacy and the prognosis. *p* < 0.05 indicates that the difference is statistically significant.

**Results:**

Postoperative hospitalization was significantly shorter in the early group than in the intermediate and late groups (9.5 ± 4.2 d vs. 11.9 ± 3.7 d vs. 13.3 ± 4.5 d, *p* < 0.05). The incidence of postoperative lung infection (2.7% vs. 6.3% vs. 8.4%), deep vein thrombosis (3.9% vs. 6.7% vs. 11.6%), stress ulcers (1.9% vs. 3.6% vs. 7.1%), and pressure ulcers (2.3% vs. 6.7% vs. 7.7%) was the lowest in the early group, followed by the intermediate group, and the highest in the late group (*p* < 0.05). In-hospital mortality was lower in the early group than in the intermediate and late groups (3.5% vs. 8.0% vs. 10.3%). In addition, at 1 month postoperatively, Harris scores were significantly higher in the early group than in the intermediate group (87.1 ± 5.3 vs. 82.2 ± 5.6, *p* < 0.001) and in the intermediate group than in the late group (82.2 ± 5.6 vs. 78.4 ± 5.0, *p* = 0.008). At 1 year postoperatively, the mortality rate was lower in the early group than in the intermediate and late groups (2.4% vs. 6.8% vs. 7.2%).

**Conclusion:**

Early surgery reduces the incidence of postoperative complications in elderly hip fracture patients, shortens hospitalization time, facilitates early recovery of hip function, and reduces mortality within 1 year after surgery.

## Introduction

Elderly hip fracture is one of the common and serious diseases in orthopedics, which is mostly caused by osteoporosis and low-energy injuries (1–3). The most common clinical types include femoral neck fractures and intertrochanteric fractures (4). As the population ages, the incidence of elderly hip fracture is increasing, and it is expected that by 2050, the annual incidence of hip fracture will approach 4.5 million cases worldwide (5). Hip fracture not only brings great pain to patients and seriously affects their quality of life and mortality, but also imposes a heavy burden on families and society (6–9). The treatment of hip fracture includes non-surgical treatment and surgical treatment, and for elderly hip fracture patients, surgical treatment is the main method at present (10–12). Surgical timing refers to the waiting time between the patient’s injury and the start of surgery, including the time of prehospital transport and the time of preoperative evaluation after admission. The choice of surgical timing has a crucial impact on surgical outcomes, the occurrence of postoperative complications, and patient prognosis (13–16). From the physiopathological point of view, the body functions of elderly patients gradually decline with age, and the compensatory capacity of various organ systems is weakened. After hip fracture, patients’ prolonged bed rest is prone to a series of complications, such as lung infection, deep vein thrombosis, pressure ulcers and so on, which will further aggravate the patients’ physical condition (17–19).

Currently, there is no uniform standard on the optimal timing of surgery for elderly hip fracture, and there is much controversy in clinical practice. Some studies believe that early surgery can effectively reduce the incidence of postoperative complications and mortality and improve the prognosis of patients (20–22). While some other studies believe that elderly hip fracture patients are older, with more comorbid underlying diseases, requiring longer preoperative preparation time, and that appropriately prolonging the preoperative waiting time is favorable to the patients’ prognosis, and that the choice of the timing of surgery has nothing to do with the patients’ postoperative mortality rate (23–25). An in-depth study of the effect of surgical timing on postoperative complications and prognosis in elderly hip fracture patients is of great clinical significance for the development of individualized and precise surgical plans. The aim of this study was to investigate the effect of surgical timing on postoperative complications and prognosis of elderly hip fracture patients through retrospective analysis, and to provide an evidence-based basis for optimizing clinical decision-making.

## Materials and methods

### Patients and study design

**Figure 1:**
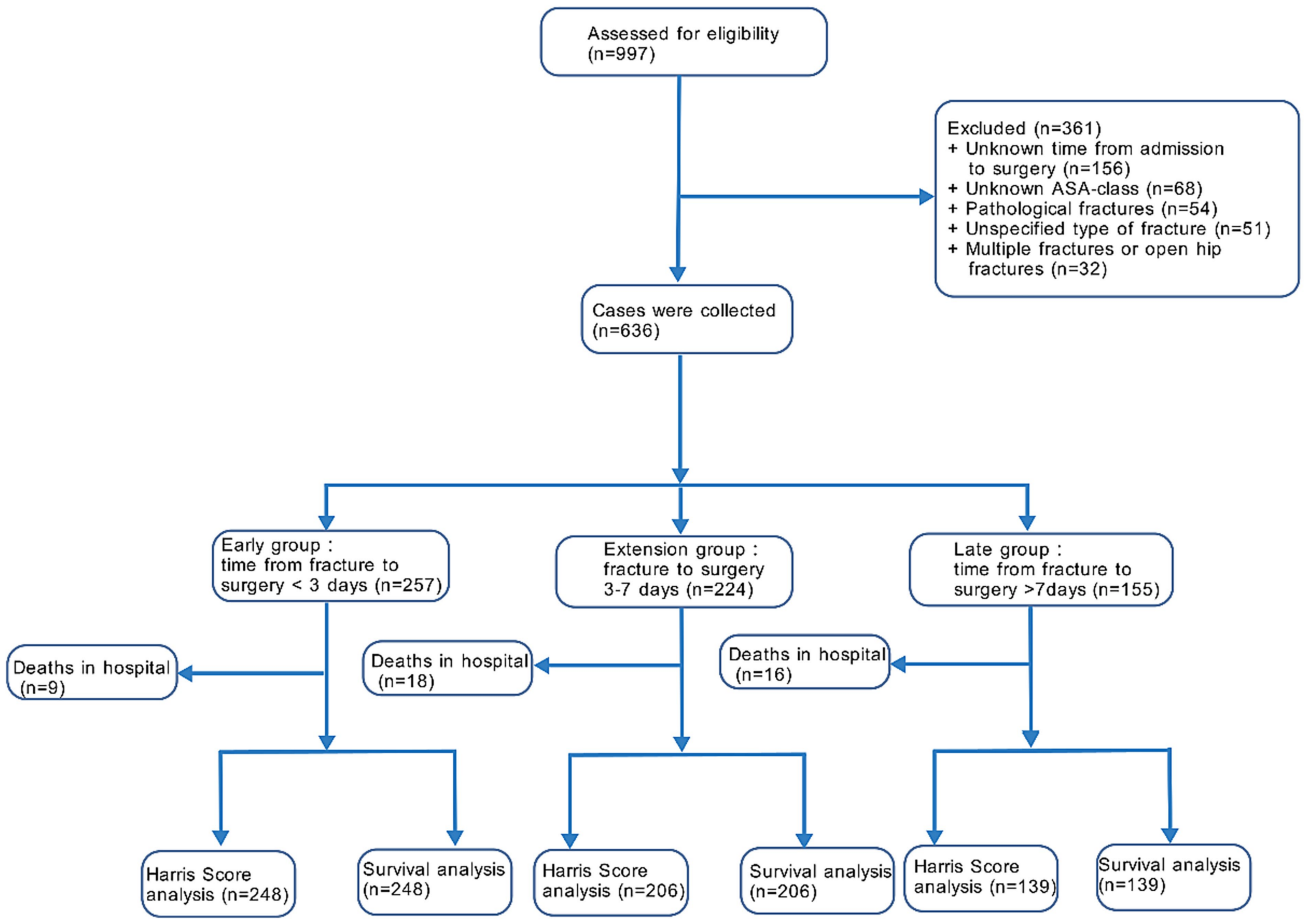
Flowchart for patients included in the study.

This retrospective cohort study included 997 consecutive patients over 65 years of age with a diagnosis of traumatic hip fracture admitted to our hospital from January 2019 to January 2024. A total of 361 patients were excluded based on the screening criteria and 636 patients were included in the final analysis. Patients were categorized into three groups based on the time from fracture onset to surgery: less than 3 days for the early group, 3–7 days for the intermediate group, and more than 7 days for the late group (Figure 1). Surgery was scheduled by the medical staff after assessing the health status of the patients. The study complied with the requirements of the Declaration of Helsinki, and all patients and their families signed an informed consent for surgery.

### Inclusion and exclusion criteria

Inclusion criteria: (1) age ≥65 years; (2) conscious; (3) fresh hip fracture diagnosed by X-ray or CT and treated surgically; (4) complete clinical data; (5) normal hip function before the fracture. Exclusion criteria: (1) pathologic fracture due to tumor metastasis; (2) multiple fractures or open hip fractures; (3) unspecified type of fracture; (4) previous ipsilateral history of previous ipsilateral hip surgery; (5) fracture of unknown ASA grade; (6) typical hemolytic reaction after blood transfusion; (7) loss to follow-up or follow-up time <12 months.

### Data collection

This study used a strict exclusion method to handle missing data during the cohort selection process, and the final cohort consisted of 636 patients with complete data. Therefore, a complete case analysis was used without estimation. Data were collected retrospectively from chart reviews by two investigators using the same study protocol, which was approved by the institutional review board. Demographic and surgery-related data included age, gender, BMI, patient comorbidities, time from fracture onset to surgery, fracture type, American society of Anesthesiologists (ASA) classification, and type of surgery. Postoperative in-hospital status included complications, ICU transfer rate, ICU retention time, and length of stay. Post hospital status included follow-up time, follow-up Harris score, and survival outcome observations.

### Outcomes

(1) Patients’ clinical baseline data. (2) General postoperative in-hospital conditions: ICU transfer rate, ICU retention time, and hospitalization time. (3) Incidence of postoperative complications: pulmonary infection: pneumonitis caused by pathogens, physicochemical factors, and other elements; urinary tract infection: positive urine culture (≥10^5^ CFU/mL) with symptoms or pyuria; incision infection: purulent drainage from the incision, or positive culture from aseptically obtained fluid, with local signs of inflammation; lower extremity deep vein thrombosis: abnormal coagulation of venous blood in the deep veins of the lower extremities, blocking the lumen and causing venous return disorders; stress ulcers: overt gastrointestinal bleeding (hematemesis or melena) confirmed by endoscopy; anemia: the number of red blood cells, or the concentration of hemoglobin in them, is lower than normal; hyperproteinemia: total serum protein > 80 g/L; delirium: acute onset of fluctuating mental status and inattention, with either disorganized thinking or altered level of consciousness; pressure ulcer: damage to the skin and subcutaneous tissue due to nutrient deficiencies, ulcers and even necrosis. (4) Details of in-hospital deaths (5) Efficacy and prognosis: Harris score at the 1st and 3rd postoperative months, total score of 100, the higher the score, the better the efficacy (26). Mortality rate of patients at 1 month, 3 months, 6 months and 1 year postoperatively.

### Statistical analysis

Data were analyzed and plotted using GraphPad Prism 8.0. Measurement data were expressed as mean ± standard deviation, and count data were expressed as [n (%)]. Comparison of measurement data between groups was performed by independent samples t-test, and comparison of count data between groups was performed by chi-square test or Fisher’s exact test, and *p* < 0.05 indicated that the difference was statistically significant.

## Results

### Baseline data

A total of 636 patients with a mean age of 76.8 years were included in the analysis, of whom 431 (67.8%) were female. Of the 636 patients, 257 (40.4%) were operated on within 3 days of fracture (early group), 224 (35.2%) were operated on within 3–7 days (intermediate group), and 155 (24.4%) were operated on after at least 7 days (late group). The 3 groups of patients were compared with respect to their age, gender, body mass index, type of fracture, ability to walk on admission, comorbidities, baseline data such as type of surgery and ASA classification were compared, and the differences were not statistically significant (*p* > 0.05) (Table 1).

**Table 1:** Comparison of the general data of the three groups of patients [*n* (%)].

Characteristic	Total (*n* = 636)	Early group (*n* = 257)	Intermediate group (*n* = 224)	Late group (*n* = 155)	*p*-value
Age (years)					0.079
65–74	196 (30.8)	86 (33.5)	68 (30.4)	42 (27.1)	
75–84	300 (47.2)	128 (49.8)	103 (46.0)	69 (44.5)	
≥85	140 (22.0)	43 (16.7)	53 (23.6)	44 (28.4)	
Sex					0.093
Male	205 (32.2)	90 (35.0)	76 (33.9)	39 (25.2)	
Female	431 (67.8)	167 (65.0)	148 (66.1)	116 (74.8)	
BMI, kg/m^2^					0.426
<18.5	105 (16.5)	35 (13.6)	43 (19.2)	27 (17.4)	
18.5–24.0	375 (59.0)	162 (63.0)	125 (55.8)	88 (56.8)	
>24.0	156 (24.5)	60 (23.4)	56 (25.0)	40 (25.8)	
Fracture type					0.062
Neck	333 (52.4)	123 (47.9)	131 (58.5)	79 (51.0)	
Intertrochanteric	303 (47.6)	134 (52.1)	93 (41.5)	76 (49.0)	
Walking ability					0.321
Can walk	299 (47.0)	116 (45.1)	102 (45.5)	81 (52.3)	
Cannot walk	337 (53.0)	141 (54.9)	122 (54.5)	74 (47.7)	
Comorbidity					0.594
Hypertension	197 (31.0)	86 (33.5)	68 (30.4)	43 (27.7)	
Diabetes mellitus	89 (14.0)	32 (12.5)	42 (18.8)	15 (9.7)	
COPD	64 (10.1)	20 (7.8)	31 (13.8)	13 (8.4)	
Heart failure	27 (4.2)	12 (4.7)	9 (4.0)	6 (3.9)	
IHD	16 (2.5)	6 (2.3)	7 (3.1)	3 (1.9)	
CKD	12 (1.9)	5 (1.9)	3 (1.3)	4 (2.6)	
Rheumatoid	9 (1.4)	5 (1.9)	2 (0.9)	2 (1.3)	
Surgery type					0.088
Fixation	209 (32.9)	88 (34.2)	62 (27.7)	59 (38.1)	
Replacement	427 (67.1)	169 (65.8)	162 (72.3)	96 (61.9)	
ASA					0.196
I-II	364 (57.2)	156 (60.7)	128 (57.1)	80 (51.6)	
III-IV	272 (42.8)	101 (39.3)	96 (42.9)	75 (48.4)	

BMI, body mass index; COPD, chronic obstructive pulmonary disease; IHD, ischemic heart disease; CKD, chronic kidney disease; ASA, American society of Anesthesiologists.

### Post-operative conditions

All patients underwent surgery successfully. After surgery, the number of ICU transfers was 8 (3.6%) and 12 (7.7%) in the intermediate and late groups, respectively, while the number of ICU transfers in the early group was only 5 (1.9%). ICU retention time in the early group was significantly shorter than that in the intermediate group (1.6 ± 0.4 d vs. 2.6 ± 0.3 d, *p* < 0.001), whereas the difference in ICU retention time between the intermediate group and the late group was not statistically significant (2.6 ± 0.3 d vs. 2.9 ± 0.4 d, *p* = 0.070). In addition, as the preoperative waiting time increased, patients’ postoperative hospitalization time increased, and the hospitalization time in the early group was significantly shorter than that in the intermediate group (9.5 ± 4.2 d vs. 11.9 ± 3.7 d, *p* = 0.047), whereas there was no statistically significant difference in the comparison of postoperative hospitalization time in the intermediate group versus the late group (11.9 ± 3.7 d vs. 13.3 ± 4.5 d, *p* = 0.082) (Table 2).

**Table 2:** Comparison of postoperative conditions among the three groups [*n* (%), (mean ± SD)].

Classification	Number	ICU transfer [*n* (%)]	ICU retention time (d)	Length of hospitalization (d)
Early group	257	5 (1.9)	1.6 ± 0.4	9.5 ± 4.2
Intermediate group	224	8 (3.6)	2.6 ± 0.3	11.9 ± 3.7
Late group	155	12 (7.7)	2.9 ± 0.4	13.3 ± 4.5
χ^2^/ t		8.72	5.62 / 1.92	2.05 / 1.79
*p* value		0.013	0.001^a^ / 0.070^b^	0.047^c^ / 0.082^d^

a, Comparison of ICU retention time between the early group and the intermediate group; b, Comparison of ICU retention time between the intermediate group and the late group; c, Comparison of hospitalization time between the early group and the intermediate group; d, Comparison of hospitalization time between the intermediate group and the late group.

### Complications

The difference in the incidence of postoperative urinary tract infection, incision infection, anemia, hyperproteinemia, and delirium was not statistically significant in the three groups (*p* > 0.05). And the postoperative pulmonary infection (2.7% vs. 6.3% vs. 8.4%, *p* = 0.035), deep vein thrombosis (3.9% vs. 6.7% vs. 11.6%, *p* = 0.010), stress ulcers (1.9% vs. 3.6% vs. 7.1%, *p* = 0.029), and pressure sores (2.3% vs. 6.7% vs. 7.7%, *p* = 0.025) all showed an increasing trend in incidence with increasing preoperative waiting time, with the lowest in the early group, the second highest in the intermediate group, and the highest in the late group, and the trends were all statistically significant (Table 3).

**Table 3:** Comparison of the complications of the three groups of patients [*n* (%)].

Classification	Early group (*n* = 257)	Intermediate group (*n* = 224)	Late group (*n* = 155)	χ^2^	*p* value
Lung infection	7 (2.7)	14 (6.3)	13 (8.4)	6.69	0.035*
Urinary tract infection	4 (1.6)	7 (3.1)	4 (2.6)	1.32	0.516
Incision infection	2 (0.8)	6 (2.7)	5 (3.2)	3.59	0.166
Lower extremity deep vein thrombosis	10 (3.9)	15 (6.7)	18 (11.6)	9.15	0.010*
Stress ulcers	5 (1.9)	8 (3.6)	11 (7.1)	7.10	0.029*
Anemia	14 (5.4)	18 (8.0)	13 (8.4)	1.76	0.416
Hyperproteinemia	9 (3.5)	13 (5.8)	8 (5.2)	1.50	0.472
Delirium	3 (1.2)	6 (2.7)	6 (3.9)	3.22	0.200
Pressure ulcer	6 (2.3)	15 (6.7)	12 (7.7)	7.35	0.025*

*p < 0.05.

### Cause of death in hospital

The total number of in-hospital deaths in the 3 groups was 43 (6.8%), including 9 (3.5%) in the early group, 18 (8.0%) in the intermediate group, and 16 (10.3%) in the late group. The top three causes of death were pneumonia in 9 cases (20.9%), heart failure in 6 cases (14.0%) and arrhythmia in 6 cases (14.0%), in that order. No one in the early group died of aortic aneurysm rupture, and there were more deaths from pneumonia and heart disease (heart failure and arrhythmia) in the intermediate and late groups than in the early group (Table 4).

**Table 4:** Details of cause of death in hospital [*n*].

Cause of death	Early group (*n* = 9/257)	Intermediate group (*n* = 18/224)	Late group (*n* = 16/155)	Total (*n* = 43/636)
Pneumonia	2	4	3	9
Pulmonary infarction	1	2	2	5
Cerebral infarction	2	2	1	5
Cardiac failure	1	3	2	6
Arrhythmia	1	3	2	6
Renal failure	1	1	2	4
Malignant neoplasm	1	2	2	5
Dissecting aortic aneurysm	0	1	2	3

### Efficacy and prognosis

**Figure 2:**
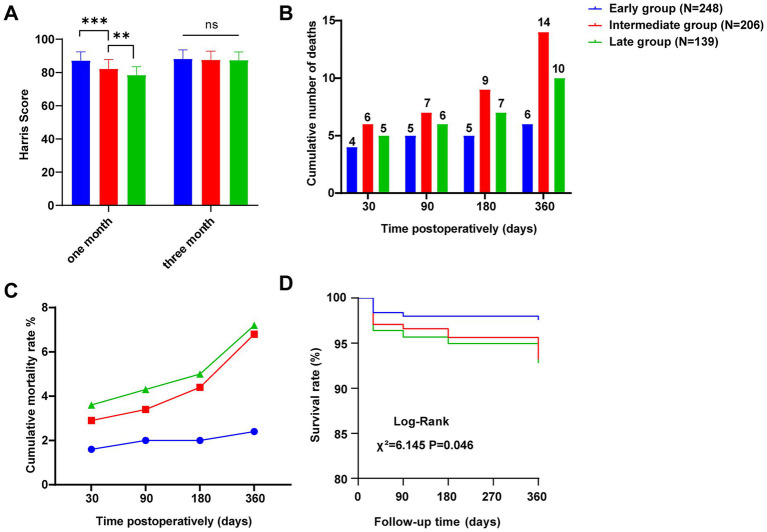
Efficacy and prognosis. **(A)** Harris scores at postoperative months 1 and 3. **(B)** Cumulative number of patient deaths at 1 month, 3 months, 6 months and 1 year postoperatively. **(C)** Cumulative mortality at 1 month, 3 months, 6 months and 1 year after surgery. **(D)** Survival curves of the three groups. **p < 0.01, ***p < 0.001.

Patients in all 3 groups received at least 1 year of postoperative follow-up. Harris scores tended to decrease at 1 month postoperatively with increasing preoperative waiting time, and were significantly higher in the early group than in the intermediate group (87.1 ± 5.3 vs. 82.2 ± 5.6, *p* < 0.001), and in the intermediate group than in the late group (82.2 ± 5.6 vs. 78.4 ± 5.0, *p* = 0.008), with no significant difference between the 3 groups at 3 months postoperatively (*p* > 0.05) (Figure 2A). After excluding the number of in-hospital deaths, the cumulative number of deaths at 1 year postoperatively in 248 patients in the early group was 6, with a cumulative mortality rate of 2.4%, the cumulative number of deaths at 1 year postoperatively in 206 patients in the intermediate group was 14, with a cumulative mortality rate of 6.8%, and the cumulative number of deaths at 1 year postoperatively in 139 patients in the late group was 10, with a cumulative mortality rate of 7.2% (Figure 2B). At 1 year postoperatively, the trend of increasing mortality was significantly higher in the intermediate group than in the early group (*p* = 0.022), whereas the difference in trend with the late group was not statistically significant (*p* = 0.906) (Figure 2C). The survival curves showed that the one -year survival rates of the early, intermediate and late groups were 97.6, 93.2 and 92.8%, respectively, and the differences between the three groups were statistically significant (*p* = 0.046) (Figure 2D).

## Discussion

The results of this study showed that the early group presented advantages in several aspects. The early group had the lowest ICU transfer rate of 1.9%, which was significantly lower than the intermediate group (3.6%) and the late group (7.7%), and the ICU retention time in the early group was also significantly shorter than that in the intermediate group (*p* < 0.001), suggesting that early surgery may relieve the pain and discomfort caused by the fracture more quickly, and lead to faster stabilization of the patient’s condition, which would in turn reduce the necessity of ICU intervention. In addition, the postoperative hospitalization time was significantly shorter in the early group than in the intermediate group (*p* = 0.047). Shorter hospitalization time means that patients can return to their daily lives more quickly, reducing the psychological and physical burdens of hospitalization, as well as reducing medical costs and the risk of hospital infection. There are many reasons for long preoperative waiting times in clinical practice, with multidisciplinary consultation and evaluation being the main reason for surgical delays, and other reasons such as inability to schedule surgeries on holidays, limited emergency surgical suites, lack of patient willingness to undergo surgery, and anticoagulant withdrawal times (27–29). Long preoperative waiting time may result in the loss of optimal surgical timing. In this study, we found that the incidence of postoperative pulmonary infection, deep vein thrombosis, stress ulcers and pressure ulcers showed an increasing trend with the intermediate of preoperative waiting time, and the incidence of complications in the early group was significantly lower than that in the intermediate and late groups (*p* < 0.05). This may be related to the fact that early surgery reduces the time the patient spends in bed for a long period of time. Prolonged bed rest is an important risk factor for complications such as pulmonary infection and deep vein thrombosis. Elderly patients’ physical functions decline, and prolonged bed rest will make the lung secretions not discharged well, which is easy to cause lung infections. At the same time, bed rest leads to slow venous blood return in the lower limbs, increasing the risk of deep vein thrombosis (30). Early surgery allows patients to move around as early as possible, reducing these risks (31, 32).

One study found a 1.1-fold increase in in-hospital mortality for every 24 h increase in preoperative waiting time (33). In addition, another study showed that Asian elderly hip fracture patients undergoing surgery with a preoperative waiting time of more than 36 h had an increased incidence of postoperative pneumonia, myocardial infarction, and heart failure (34). The results of this study showed that in-hospital mortality was 3.5, 8.0 and 10.3% in the early, intermediate and late groups, respectively. The main causes of death were pneumonia, heart failure and arrhythmia, with more deaths due to pneumonia and heart disease in the intermediate and late groups than in the early group. It is suggested that early surgery may help to reduce the risk of in-hospital deaths in patients, especially deaths due to infections and cardiac diseases. Regarding postoperative functional recovery, the Harris score of the early group was significantly higher than that of the intermediate and late groups at 1 month after surgery (*p* < 0.001), indicating that early surgery is favorable to the early functional recovery of patients. Early surgery enables patients to start rehabilitation exercises as soon as possible, avoiding muscle atrophy and joint stiffness caused by prolonged bed rest, thus better restoring the function of the hip joint and improving the quality of life. However, at 3 months after surgery, the difference in Harris scores among the three groups was no longer significant (*p* > 0.05), which may be related to the individual differences of patients in the process of postoperative rehabilitation, and the functional recovery of some patients with intermediate or late surgery can also reach a better level after active rehabilitation. A retrospective study found that there was no difference between early surgery and intermediate surgery in terms of mortality within 30 days after surgery (35). The results of this study showed that there was no significant difference in mortality rates among the three groups of elderly hip fracture patients at 1 month postoperatively, but at 1 year postoperatively, the mortality rate of the early group was 2.4%, which was significantly lower than that of the intermediate group (6.8%) and the late group (7.2%), suggesting that early surgery can improve the prognosis of the patients to a certain degree and reduce the mortality rate within 1 year postoperatively. It is worth noting that the difference in mortality rate between the late group and the intermediate group in this study was not significant, which may be related to a variety of factors, such as the patients’ underlying diseases and postoperative care.

Although the results of this study support the idea of early surgery, it should also be noted that elderly patients are often comorbid with a variety of underlying diseases, and the overall condition of the patient needs to be adequately assessed to ensure that the patient is able to tolerate the surgery before performing early surgery. For some patients with severe cardiovascular and respiratory diseases, surgery may need to be appropriately delayed to optimize preoperative status and reduce surgical risk. In clinical practice, the optimal timing of surgery should be determined individually based on the patient’s specific condition, taking into account the patient’s general condition, fracture type, comorbidities, and other factors, weighing the pros and cons of early surgery and delayed surgery, and formulating a surgical plan that is most suitable for the patient.

### Limitations

This study is a retrospective cohort study, which may have some selection bias and information bias. Patients in better condition may be selected for early surgery, which could bias the results in favor of this group of patients. When patients were grouped, although they were grouped according to the time from fracture to surgery, there may have been certain unmeasured or under considered confounding factors, such as the patient’s family economic status and the accessibility of healthcare resources, which may affect the choice of the timing of surgery and the patient’s prognosis. Unadjusted comparisons of baseline characteristics showed a significant benefit of early surgery, but the lack of multivariate regression or propensity score analyses prevented full consideration of all potential confounding influences. In addition, the sample size of the study was relatively limited, and some of the data in the study relied on medical records, which may have been incomplete or inaccurate, thus affecting the results of the study to some extent. Future studies can carry out prospective randomized controlled trials to randomly group elderly hip fracture patients according to the timing of surgery to more accurately assess the impact of different timing of surgery on postoperative complications and prognosis. At the same time, the research indexes can be further refined, such as analyzing different types of fractures (femoral neck fracture, intertrochanteric fracture, etc.) separately, to explore whether there is a difference in the impact of surgery timing on patients with different types of fractures. In addition, new technologies such as biomarkers and genetic testing can be combined to study in depth the relationship between surgical timing and patients’ body functions, inflammatory response, immune function, etc., to provide a basis for making more precise decisions on surgical timing. Through these in-depth studies, the treatment strategy of elderly hip fracture patients can be further optimized to improve the treatment effect and quality of life of patients.

## Conclusion

Compared with intermediate and late surgery, early surgery can reduce the incidence of postoperative complications, shorten hospital stay, promote early recovery of hip function, and reduce mortality within 1 year after surgery in elderly hip fracture patients.

## Data Availability

The raw data supporting the conclusions of this article will be made available by the authors, without undue reservation.
